# Molecular characterization of enteroviruses circulating among pigs and goats in two Central African countries, Cameroon and the Central African Republic

**DOI:** 10.1099/acmi.0.000886.v3

**Published:** 2025-01-08

**Authors:** Abdou Fatawou Modiyinji, Marie-Line Joffret, Marina Prisca de Marguerite Nombot-Yazenguet, Marie-Claire Endengue Zanga, Serge Sadeuh-Mba, Richard Njouom, Maël Bessaud

**Affiliations:** 1 Service de Virologie, Centre Pasteur du Cameroun, Yaoundé, Cameroon; 2 Virus Sensing and Signaling Unit, CNRS UMR 3569, Institut Pasteur, Université de Paris Cité, Paris, France; 3 Laboratoire associé au Centre national de référence entérovirus/paréchovirus, Paris, France; 4 Laboratoire des Hépatites Virales, Institut Pasteur de Bangui, Bangui, Central African Republic

**Keywords:** Cameroon, Central African Republic, enterovirus, *Enterovirus species G*, EV-G, zooanthroponosis, *Enterovirus geswini*

## Abstract

To date, data on animal enteroviruses (EVs) are scarce, especially in Central Africa. The aim of this study was to characterize EVs among pigs and goats in Cameroon and the Central African Republic (CAR). A total of 226 pig and goat faecal samples collected in two previous studies carried out in Cameroon and CAR were pooled and screened with molecular assays targeting EV-Es, EV-Fs and EV-Gs. EV genomes were amplified by RT-PCR and their sequences were obtained by Illumina sequencing and *de novo* assembly. Based on the capsid sequences, 27 EV-G sequences were identified and assigned to 11 virus types, while no EV-E or EV-F was observed. Phylogenetic analysis revealed that the EV-Gs detected in Central Africa do not form specific clusters compared to EV-Gs previously reported in other continents. This suggests a worldwide circulation of EV-Gs, which is likely due to the massive international trade of live animals. One human EV, EV-C99, which belongs to the species *Enterovirus C*, was detected in pigs. This is the third detection of such an event in a similar context, reinforcing the hypothesis that some EV-Cs could be infecting pigs. Our work provides new data on the genetic diversity of EVs circulating among domestic animals in Central Africa.

## Data Summary

Genetic sequences were submitted to GenBank (accession numbers PQ043000–PQ043027).

## Introduction

Enteroviruses (EVs), members of the *Picornaviridae* family, form a large group of viruses that infect numerous mammal species. The *Enterovirus* genus currently comprises over 300 different virus types, grouped into 15 species: *Enterovirus A-L* and *Rhinovirus A-C* [1]. EV genome contains a long ORF flanked by two untranslated regions (UTRs). The ORF encodes a polyprotein containing the four structural proteins that form the capsid (VP1 to VP4) and all the non-structural proteins. Among the four proteins that compose the virus capsid, VP1 bears the major neutralizing epitopes. Therefore, the original classification of EVs into serotypes is broadly consistent with the classification based on the VP1-encoding sequences. The VP1-encoding region is thus widely used to determine the virus types of EV field strains through molecular characterization [2,3].

Members of the species *Enterovirus E*, *F* and *G* (EV-E, EV-F and EV-G) are associated with animal diseases affecting the livestock industry. The species *Enterovirus E* and *Enterovirus F* formed initially a unique species called *Bovine Enterovirus* [4,5]. EV-Es and EV-Fs have mainly been reported in cattle, but they are able to infect a wide range of mammals, including dolphins and primates [5,11]. EV-Es and EV-Fs have been sampled mostly in Asia, with a few cases in the USA, Egypt, Nigeria, Brazil, Europe, Australia, Bangladesh and New Zealand. The species *Enterovirus G* was previously referred to as *Porcine enterovirus* because its first members were sampled in suidae. It was subsequently discovered that some members of this species circulate in sheep and goats [5]. EV-Gs have been sampled in Europe, Asia, the Americas and Africa. To date, data on EVs that circulate in herd animals are scarce in Central Africa [12,15]. This geographic region seems to harbour a particular ecosystem of EVs circulating in humans, with a relatively high proportion of EV-Cs [12,16,19] compared to other regions where these viruses are seemingly less abundant. Only two publications have reported the detection of EVs in farm animals in Central Africa, one in Gabon [15] and one in the Central African Republic (CAR) [12]. Both revealed EV-Gs, some of which were genetically close to EV-Gs sampled in other continents. For instance, some EV-G4s, EV-G8s and EV-G17s sampled in CAR were very close to viruses sampled in China in the VP1-encoding region [12]. Other EV-Gs sampled in CAR belonged to a hitherto unknown virus type that was named EV-G28 [12]. Neither EV-E nor EV-F was detected in these two studies, but one EV-E was isolated from a sewage-contaminated water sample collected in Nigeria in 2017 [13].

The aim of this study was to provide additional data on the genetic diversity of EVs among domestic animals (pigs and goats) in Cameroon and CAR. Pools of stool samples were molecularly screened with assays targeting EV-Es, EV-Fs and EV-Gs. The EV genomes thus detected were sequenced by Illumina techniques and compared to genomes available in public databases.

## Methods

### Study sites and sample collection

We performed a retrospective study by analysing animal stool samples previously collected as part of two studies investigating the presence of hepatitis E virus in pigs and goats in Cameroon and CAR [20,21]. In Cameroon, stools were collected in slaughterhouses in Yaoundé (*Marché huitième*) and Douala (*Marché des chèvres*) after pig slaughter between February 2017 and September 2018. In CAR, anorectal swabs were taken from pigs and goats on farms in five districts of Bangui (second, fourth, sixth, seventh and eighth) between January and October 2021. These stool samples were stored at the Virology Unit of the *Centre Pasteur du Cameroun* at −80 °C and analysed in the present study to detect and characterize animal EVs.

### RNA extraction and molecular screening

Faecal samples were diluted in PBS and clarified by centrifugation following the guidelines of the Polio Laboratory Manual [22]. After clarification, individual animal samples were pooled according to their respective animal species and collection sites before molecular screening. RNAs from each pooled suspension were extracted using the ZYMO_RESEARCH® Kit according to the manufacturer’s instructions. EV RNAs were detected by real-time RT-PCR using a protocol recently published [12]. Primers and probes were designed to target conserved nucleotide sequences in the 5′UTR of EV-E, EV-F and EV-G. Because of the genetic variability in the 5′UTR, two probes were designed: one targeting the 5′UTRs of EV-E, EV-F and some EV-Gs (assay A) found in goats and sheep and the second targeting most EV-Gs (assay B).

Detection of EV RNA was performed using SuperScript™ III One-Step RT-PCR System with Platinum Taq (Life Technologies Corporation, USA) in a final volume of 20 µl by mixing 2 µl of extracted RNA with 0.4 µl of SuperScript RT/Platinum III Taq Mix, 10 µl of Reaction (2X), 10 pmol of each primer, 5 pmol of each probe and 4.6 µl of nuclease-free water. The thermocycler profile was 45 °C for 15 min, 95 °C for 2 min, followed by 45 cycles of PCR (95 °C for 15 s and 60 °C for 30 s).

### EV genome amplification and sequencing

For amplifying the EV genomes prior to sequencing, two overlapping fragments were amplified using generic primers already described [12]. The first half of the genomes was amplified using primers targeting conserved genetic sequences in the 5′UTR and in the *cis*-replicating element (cre) located within the 2C-encoding region (Table 1). They were designed based on EV-E, EV-F and EV-G genetic sequences retrieved from GenBank. To obtain cDNA, a reverse transcription step was performed using the Maxima H Minus First Kit Strand cDNA Synthesis Kit (Thermo Scientific, ref K1652) in a volume of 20 µl with 4 µl of extracted RNA, 10 nM of each dNTP, 1 µl of HeptaN (0.5 µg.µl^−1^) and 14 µl of nuclease-free water. The mixture was incubated at 65 °C for 5 min and then on ice for 2 min; after adding 4 µl of RT buffer (5X) and 1 µl of Maxima H minus enzyme Mix, incubation was performed at 50 °C for 30 min and 70 °C for 5 min. PCR was performed using cDNA as a template and the Phusion High-Fidelity DNA polymerase kits (Thermo Scientific) as previously described. The reaction mixture was as follows: 2 µl of cDNA, 5 µl of Phusion HF Buffer (5X), 10 nM µl of each dNTP, 10 pmol of each primer (EV-EFG-271-F/EV-EFG-4441-R) and 0.5 µl of enzyme. The thermocycler profile was 98 °C for 30 s followed by 35 cycles of amplification (98 °C for 10 s, 50 °C for 30 s and 72 °C for 2 min) and a final extension step at 72 °C for 10 min. If no band appeared on the agarose gel at the expected size, two nested PCRs were performed in parallel with the primer pairs EV-EF-381-F/EV-EF-4357-R and EV-G-491-F/EV-G-4294-R by using 2 µl of the product of the first PCR as a template under the same thermal conditions.

**Table 1.** Primers used in our study

	Name	5′-3′ sequence
**Molecular screening**		
Sense primer	EV-EFG-546-F	CTAATCCCAACCTCSGAGC
Antisense primer	EV-EFG-632-R	ACCSAAAGTAGTCTGTTCC
Probe A	EV-EF-probe	FAM-CCAGTGTTGCTACGTCGTAA-TAMRA*
Probe B	EV-G-probe	Cy5-GGYGTCGTAACGGGYAACTCTGTG-IBRQ†
**Amplification of the 5′ half of the genome**		
*First PCR*		
Sense primer	EV-EFG-271-F	GGTCAAGCACTTCTGTYTC
Antisense primer	EV-EFG-4441-R	CGDTKCTTGBTCTTGAACTG
***Nested PCR***		
Sense primer	EV-EF-381-F	CRGYGGTAGCTCTGRRDRATG
Antisense primer	EV-EF-4357-R	GCRTTYTTYCTRCARTGRTG
*Nested PCR*		
Sense primer	EV-G-491-F	GAATGCKGCTAATCCTAACC
Antisense primer	EV-G-4294-R	CCAAANADGGTYTCYTGYTG
**Amplification of the 3′ half of the genome**		
Sense primer	EV-G-4263-F	CCAACYACRGAGCAGCARG
Antisense primer	J-polyT	CAGGAAACAGCTATGACTTTTTTTTTTTTTTTTTTTTTVN

*FAM: 6-carboxyfluorescein; TAMRA: 6-carboxy-tetramethyl-rhodamine.

†Cy5: Cyanine 5; IBRQ: 3′ Iowa Black RQ.

The second half of the EV-G genomes was amplified according to the same protocol using a sense primer (EV-G-4263-F) that targets a conserved sequence within the 2C-encoding region and a reverse primer (J-polyT) that targets the polyA tail (Table 1).

After electrophoresis on an agarose gel, the products of the PCR or nested PCR with bands of the expected size were purified on a NucleoFast®_96_PCR_Plate-DNA prior to sequencing by Illumina technology [23]. Sequencing was performed using the Nextera XT DNA Library Preparation kit according to the manufacturer’s recommendations. Libraries were built using 0.1 ng of DNA with the Nextera XT DNA Library Preparation kit in a PCRmax Alpha Cycler 1 Thermal Cycler (Cole-Parmer). After purification on AMPure beads (Beckman), the libraries were controlled using the High Sensitivity D1000 assay (Agilent) on a TapeStation 4200 (Agilent). Sizing was achieved by electrophoresis on a PippinPrep System with the PippinPrep kit CDF1510 (Ozyme).

### Sequence analysis and molecular typing of EV specimens

The Illumina raw sequences were analysed using CLC Genomics software (version 22). Contigs were built *de novo* and the contigs that correspond to EV genomic sequences were flagged through a blast analysis. Molecular typing of EV strains was based on the capsid-encoding genomic region, especially the VP1-encoding sequence. The complete VP1 nucleotide sequences were aligned using the ClustalW program and phylogenetic trees were reconstructed using the mega X program [24]. The neighbour-joining algorithm was used to generate the initial tree. The percentage of replicate trees in which the associated taxa clustered together in the bootstrap test was calculated from 1000 replicates.

The nucleotide sequences of 27 EV-Gs and 1 EV-C identified in this study have been deposited in GenBank with the accession numbers PQ043000–PQ043027.

## Results

### Origin of the samples

A total of 226 animal stools were analysed in this retrospective study, including 136 collected from pigs in slaughterhouses in Cameroon (Yaoundé *n*=58 and Douala *n*=78) and 90 collected from pigs (*n*=60) and goats (*n*=30) in farms in Bangui, CAR. Individual animal samples were pooled according to their respective animal species and collection site, and we obtained 20 pools for pigs (6 pools in Yaounde, 8 pools in Douala and 6 pools in Bangui) and 3 pools for goats (only in Bangui).

### Detection of EV-Gs and one EV-C in stools and sequencing

After pooling and RNA extraction, EVs were detected by RT-PCR using a primer pair and two probes targeting the 5′UTR. The probe A (targeting the 5′UTRs of EV-E, EV-F and some EV-Gs) gave no positive result, while the probe B (targeting most EV-Gs) was positive for 12 pools from pigs, including 6 in Cameroon and 6 in CAR (Table 2).

**Table 2.** Molecular screening and RT-PCR results

Host	No. of pools	No. of positive pools by screening		No. of pools from which an amplicon was obtained by RT-PCR	
		Probe A	Probe B	5′half amplification	3′half amplification
**Cameroon**					
Pigs	14	0	6	3	1
**Central African Republic**					
Pigs	6	0	6	6	1
Goats	3	0	0	0	0

After screening by Taqman assays, two pairs of generic primers were used to perform RT-PCR and, when necessary, a subsequent nested PCR. Both primer pairs target conserved sequences flanking the capsid-encoding region: the sense primers target the 5′UTR and the antisense primers target the *cis*-replicating element (cre) located within the 2C-encoding region. The corresponding amplicons were ~3800 nt long and encompassed the whole capsid and a large fraction of the 5′UTR and P2 genomic regions. Of the 12 pools that gave positive results through molecular screening, 9 pools were positive after RT-PCR with amplicons at the expected size for each of the 9 pools (Table 2). To obtain complete genomes of animal EVs, the second half of the EV-G genomes was amplified using primers that target a conserved sequence within the 2C-encoding region and a reverse primer that targets the polyA tail. Of the 9 pools that gave a positive result through 5′half genome amplification, 2 pools were positive for 3′half genome amplification. Thus, we obtained two complete genomes of EV-G (Table 2).

After amplification, Illumina sequencing and data analyses, nucleotide sequences corresponding to the complete VP1 capsid gene were considered for typing. The number of VP1 sequences per pool of animal stools ranged from 1 to 7. Overall, 28 VP1 sequences were identified, 27 of which belonged to the species EV-G and 1 to the species EV-C. No EV-E or EV-F was characterized in the pig and goat samples analysed in our study.

**Fig. 1.**
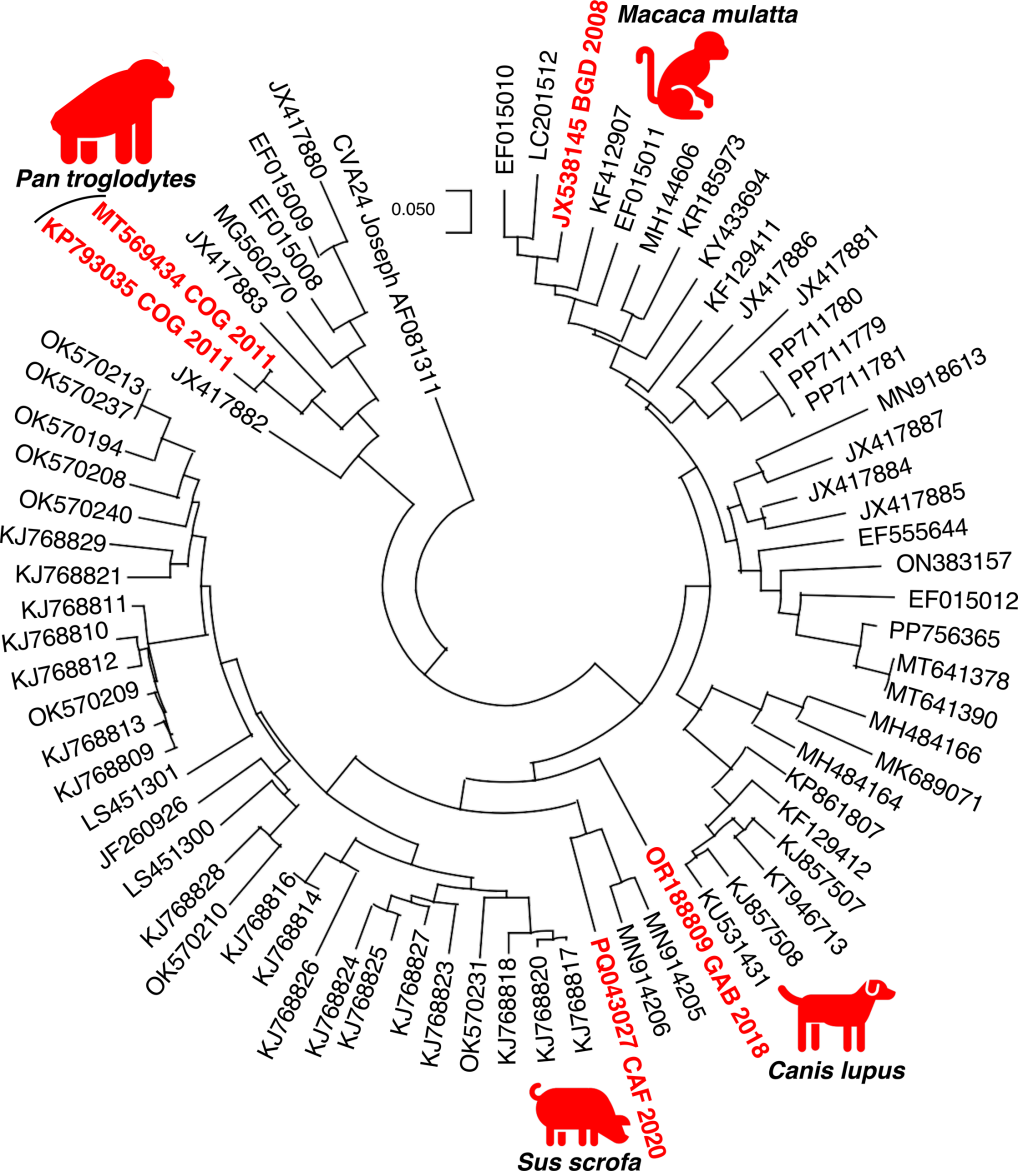
Phylogenetic relationships between EV-C99s, based on partial VP1-encoding region (nucleotide positions 2636–2932 according to the numbering of the strain GA84-10639, which is the EV-C99 prototype). The viruses are named by their respective GenBank accession numbers. Sequences from non-human stools are indicated in red, with the country (ISO 3166–1 alpha-3 codes) and year of sampling and the name of the corresponding animal species. The sequence of CVA24 strain Joseph was introduced as outgroup for rooting of the tree.

The EV-C came from a pool of pig samples collected in CAR and was an EV-C99. EV-C99s were initially not distinguished from members of another virus type named coxsackievirus (CV) A24. They were assigned to a new virus type, EV-C99, based on pairwise identities, similarity plots and phylogenetic analyses [25]. EV-C99s have been sampled worldwide and are particularly abundant in children in sub-Saharan Africa [12,26,28]. Only a few EV-C99 specimens have been reported from stools of non-human sources: one came from a dog in Gabon [15] and three from non-human primates in Bangladesh [6] and the Republic of the Congo [29,30]; to our knowledge, our study constitutes the first report of an EV-C99 in a pig. In the VP1 region, which constitutes the only data available about two EV-C99s from non-human sources (one from a dog and one from a macaque), the viruses from animal samples did not share noteworthy relationships, as they fell into different branches with numerous EV-C99 from humans (Fig. 1). In the 2A-2C region, the EV-C99 specimen detected in pig stools in this study displayed a relatively low nucleotide homology (< 73.1%) with those previously detected in stools from chimpanzees in the Republic of the Congo. In this genomic region, it was also relatively distant from two EV-Cs (one CVA17 and one CVA24) previously found in the stools of pigs sampled in CAR [12], with which it shares less than 73.1% of nucleotide homology. These observations did not reveal any common genetic features between them, which is not in favour of a common origin of the EV-Cs sporadically detected in stools of non-human mammals.

**Fig. 2.**
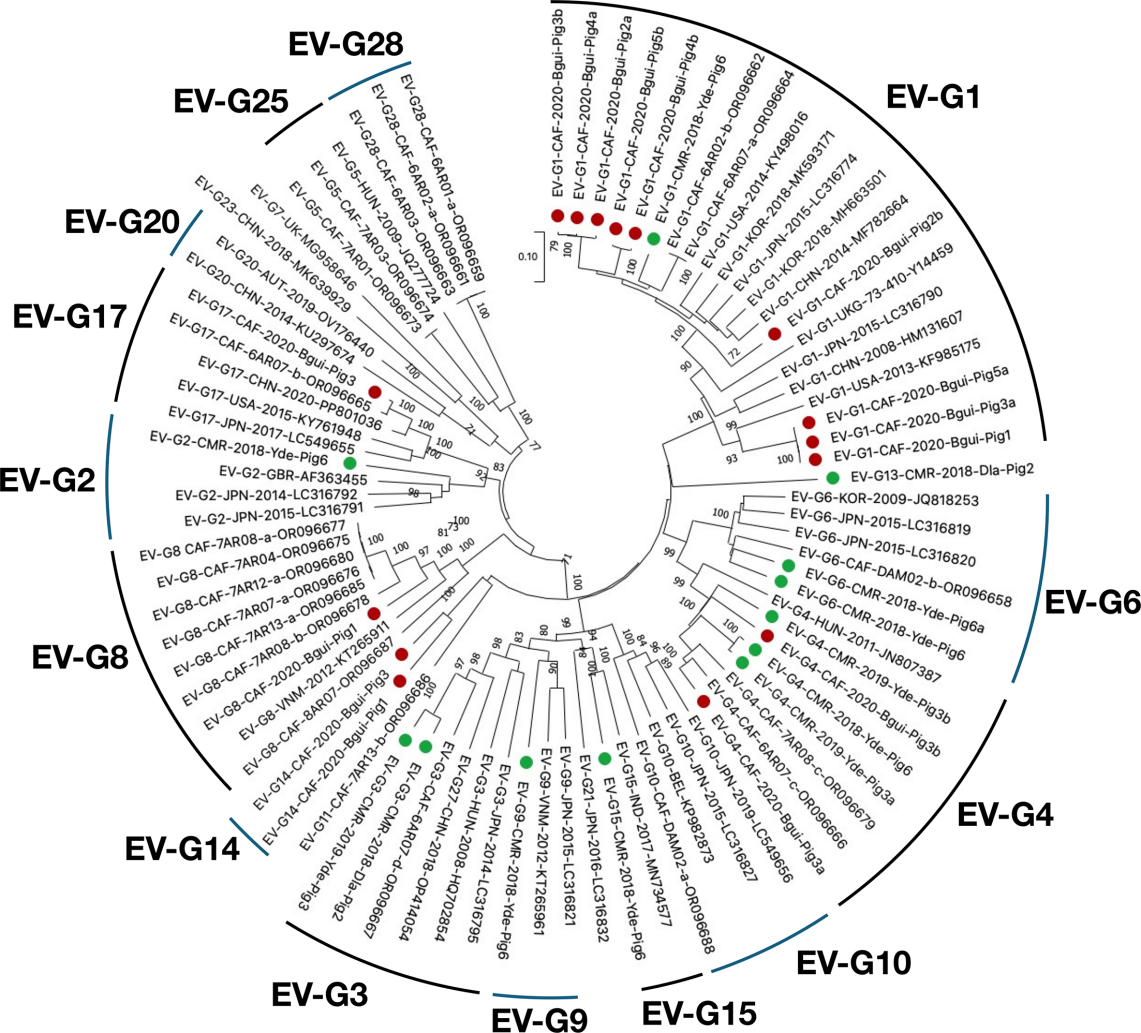
Phylogenetic relationships among Cameroon EV-Gs, CAR EV-Gs and other specimens available in public databases, based on the VP1-encoding region. Sequences from Cameroon are indicated by green circles, while those from CAR are indicated by red circles. The other sequences are named by using their respective genotype, country (ISO 3166–1 alpha-3 codes), year of sampling and GenBank accession number. Bootstrap values are indicated if >70%.

**Fig. 3.**
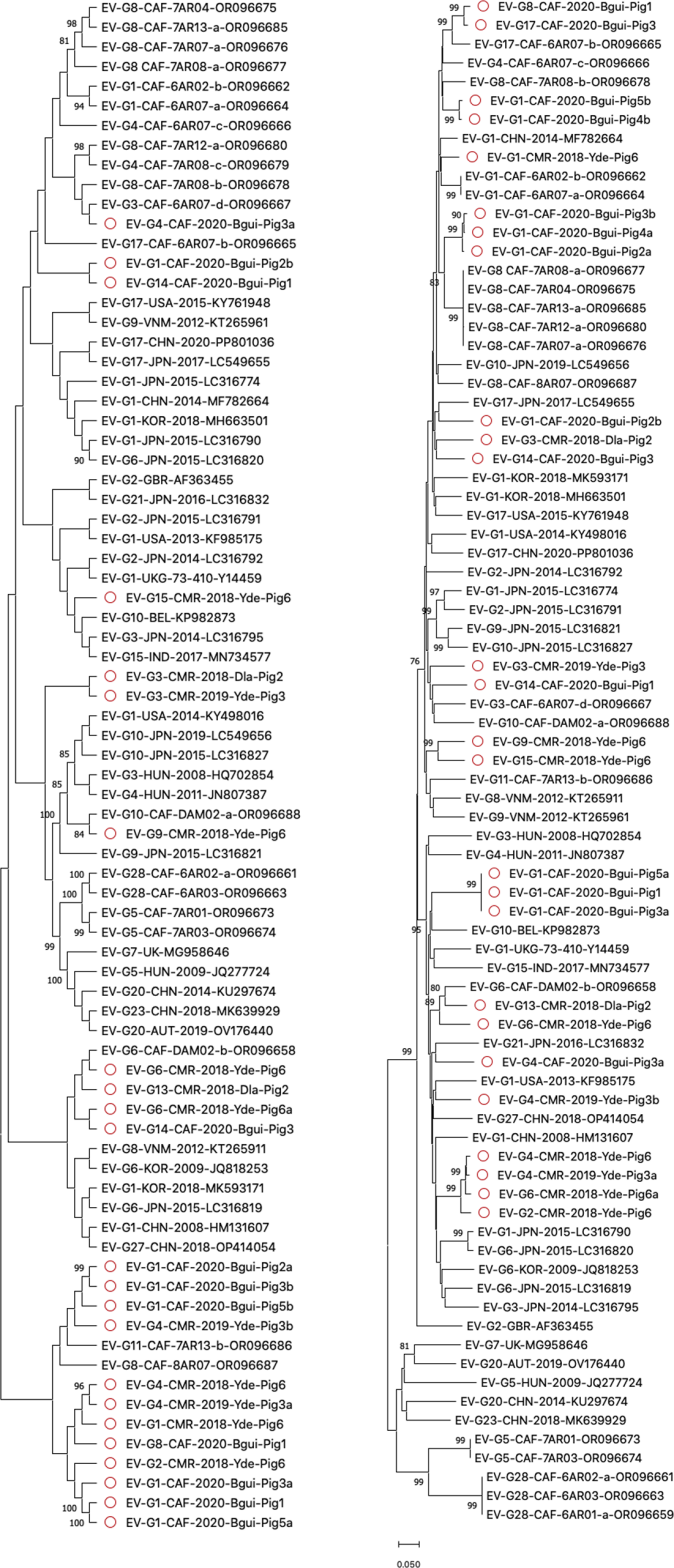
Phylogenetic relationships between EV-Gs found in our study and other specimens available in public databases, based on the 5′UTR (left) and 2A-2B (right) regions. The sequences found in our study are indicated by red circles. The other sequences are named by using their respective genotype, country (ISO 3166-1 alpha-3 codes), year of sampling and GenBank accession number. Bootstrap values are indicated if ≥70%.

EV-Gs have been classified into 28 virus types (EV-G1 to EV-G28) based on VP1 relationships higher than 75% nt identity [13]. Our 27 EV-Gs were assigned to 11 virus types already described: EVG-1, G2, G3, G4, G6, G8, G9, G13, G14, G15 and G17 (Fig. 2). All these virus types have already been reported in different Asian countries, including China, Vietnam, Thailand, India, South Korea and Japan [31,35]. Most of them have also been observed in Europe [36,37] and/or in the USA [38,39]. In order to investigate the genetic relationships between the EV-Gs detected in our study and specimens that have been previously reported, we used two genomic regions independently (the 5′UTR and 2A-2B regions), which surround the capsid coding region. Among the 27 EV-G contigs of our dataset, 23 and 26 spanned the 5′UTR and the 2A-2B region, respectively. Genetic sequences with high similarity were found using the Basic Local Alignment Search Tool (blast) and phylogenetic trees were drawn. The phylogenetic analyses of our sequences based on both regions showed that sequences do not cluster by virus types, indicating that intertypic recombination takes place between EV-Gs on either side of the capsid-encoding region (Fig. 3). Interestingly, EV-Gs from Cameroon and CAR do not form topotypes specific to Central Africa but fell into different branches, some of which contained EV-Gs sampled in other geographic regions. For instance, in the 5′UTR, the virus EV-G15 CMR-2018-Yde-Pig6 was relatively close to EV-Gs sampled in Belgium, Japan and India (Fig. 3, left tree). Similarly, in the 2A-2B region, some EV-Gs of this study shared a relatively recent ancestor with viruses sampled in China, Japan, Vietnam, Belgium, the USA and Korea (Fig. 3, right tree). These observations do not support the hypothesis that farm animals living in Central Africa harbour a specific ecosystem of EV-Gs but rather suggest a widespread circulation of these viruses.

## Discussion

The aim of our study was to identify EVs circulating among domestic animals (pigs and goats) in two Central African countries, Cameroon and CAR. To our knowledge, this is the first study reporting the genetic diversity of EVs in domestic animals in Cameroon and the second in CAR after the one recently published [12]. We found EV-Gs in the animal samples but neither EV-E nor EV-F. EV-Es and EV-Fs are mostly sampled in cattle, but some specimens were previously reported from different other mammal species, including goats [10]. Previous studies in CAR and Gabon [12,15] also reported the absence of EV-Es and EV-Fs in the stools of herd animals, including cattle, which suggests that EV-Es and EV-Fs were not abundant in herd animals in this region when these different studies were carried out (from Feb 2017 to Aug 2022).

Our study constitutes the second report of EV-Cs in the stools of pigs, as two human EV-Cs (CVA17 and CVA24) were recently identified in pigs in CAR [12]. To our knowledge, the ability of EV-Cs to infect animals has never been demonstrated. The hypothesis of a passive carriage occurring through consumption of contaminated water, faeces or food cannot be ruled out. Nonetheless, previous studies had demonstrated sustained transmission in pigs of one lineage of CV-B4 and one lineage of CV-B5, following the crossing of the species barrier by these two human members of the species *Enterovirus B* [5,40, 41]. The low number of EV-Cs detected in pig samples in this study and in previous ones and the absence of genetic links between them do not suggest an active transmission of EV-Cs from pig to pig, but further investigations are needed to determine whether pigs could constitute efficient reservoirs for these viruses.

Our study identified viruses belonging to 11 EV-G types already described. Whichever the genomic region, the EV-Gs found in this study do not segregate into specific clusters. These results concur with previous observations made in CAR [12] and suggest a global circulation of EV-Gs that can be explained by the high number of live pigs imported every year by sub-Saharan countries, including Cameroon and CAR. The livestock trade contributes to the global dissemination of viruses and can lead to the transboundary spread of diseases that have a high economical and/or ecological impact, such as African swine fever that spread out from Africa and reached Europe and Asia [42].

In conclusion, this study conducted in Cameroon and CAR reported a diversity of animal EV-Gs that are genetically linked to strains sampled in other continents, while no EV-E or EV-F was detected. The presence of EV-C99 in pigs reinforces the hypothesis that domestic pigs could be infected by human EVs, but their role as an effective reservoir remains to be determined.

## Abbreviations

CAR: Central African Republic
CV: coxsackievirus
EVs: enteroviruses
RT: reverse transcription
UTRs: untranslated regions
